# Development and Validation of an RNA-Seq-Based Prognostic Signature in Neuroblastoma

**DOI:** 10.3389/fonc.2019.01361

**Published:** 2019-12-04

**Authors:** Jian-Guo Zhou, Bo Liang, Su-Han Jin, Hui-Ling Liao, Guo-Bo Du, Long Cheng, Hu Ma, Udo S. Gaipl

**Affiliations:** ^1^Department of Oncology, Affiliated Hospital of Zunyi Medical University, Zunyi, China; ^2^Department of Radiation Oncology, Universitätsklinikum Erlangen, Erlangen, Germany; ^3^Affiliated Nanjing Hospital of Chinese Medicine, Nanjing University of Chinese Medicine, Nanjing, China; ^4^Department of Orthodontics, Affiliated Stemmatological Hospital of Zunyi Medical University, Zunyi, China; ^5^College of Integrated Traditional Chinese and Western Medicine, Southwest Medical University, Luzhou, China; ^6^Department of Oncology, Affiliated Hospital of North Sichuan Medical College, Nanchong, China

**Keywords:** neuroblastoma, gene pairs, prognostic model, validation, overall survival, event-free survival

## Abstract

**Objective:** The stratification of neuroblastoma (NBL) prognosis remains difficult. RNA-based signatures might be able to predict prognosis, but independent cross-platform validation is still rare.

**Methods:** RNA-Seq-based profiles from NBL patients were acquired and then analyzed. The RNA-Seq prognostic index (RPI) and the clinically adjusted RPI (RCPI) were successively established in the training cohort (TARGET-NBL) and then verified in the validation cohort (GSE62564). Survival prediction was assessed using a time-dependent receiver operating characteristic (ROC) curve and area under the ROC curve (AUC). Functional enrichment analysis of the genes was conducted using bioinformatics methods.

**Results:** In the training cohort, 10 gene pairs were eventually integrated into the RPI. In both cohorts, the high-risk group had poor overall survival (OS) (*P* < 0.001 and *P* < 0.001, respectively) and favorable event-free survival (EFS) (*P* = 0.00032 and *P* = 0.06, respectively). ROC curve analysis also showed that the RPI predicted OS (60 month AUC values of 0.718 and 0.593, respectively) and EFS (60 month AUC values of 0.627 and 0.852, respectively) well in both the training and validation cohorts. Clinicopathological indicators associated with prognosis in the univariate and multivariate regression analyses were identified and added to the RPI to form the RCPI. The RCPI was also used to divide populations into different risk groups, and the high-risk group had poor OS (*P* < 0.001 and *P* < 0.001, respectively) and EFS (*P* < 0.05 and *P* < 0.05, respectively). Finally, the RCPI had higher accuracy than the RPI for the prediction of OS (60 month AUC values of 0.730 and 0.852, respectively) and EFS (60 month AUC values of 0.663 and 0.763, respectively) in both the training and validation cohorts. Moreover, these differentially expressed genes may be involved in certain NBL-related events.

**Conclusions:** The RCPI could reliably categorize NBL patients based on different risks of death.

## Introduction

Neuroblastoma (NBL) is a pediatric cancer arising from neural crest precursor cells of the sympathetic nervous system. According to the World Health Organization, childhood cancer is relatively rare, accounting for only 0.5–4.6% of all cancers. Moreover, NBL is the most prevalent cancer in children after leukemia and brain cancer (1). NBL is an aggressive cancer and accounts for more than one in five cancer-related deaths in children (2), and those between 18 months and 5 years old are the most severely affected. The diagnosis depends mainly on histopathological features accompanied by elevated urinary catecholamine concentrations rather than relying solely on routine tests, such as laboratory tests, computed tomography, or magnetic resonance imaging (3, 4). NBL diagnosed in children often metastasizes, causing accelerated cancer-related death despite harsh therapies (5). In addition, current treatments for NBL include rigid chemoradiotherapies, which often leave lifelong complications for surviving patients. The recognition of more sensitive and specific signatures for therapy and outcomes is required and is expected to result in a better choice of risk-related therapy. The discovery of a preferable signature could improve the prognosis of high-risk patients and reduce the burden of constant side effects in surviving children.

There is no doubt that the tumor microenvironment substantially contributes to the biology of NBL (6). Moreover, many genes are involved in the initiation and progression of NBL, such as MYCN (7), ALK (8, 9), and LMO1 (10, 11). When such a large number of genes are evaluated as outcome signatures, it is highly possible to detect an association between gene expression and prognostic classification. The expression imbalance in certain gene pairs might play a more important role than individual differentially expressed genes (12). Moreover, compared to predictors based on individual genes, gene pair-based predictors are more robust to normalization and have better predicting or classifying accuracy (13). The gene pair-based approach has an important advantage in that the score is calculated based entirely on the gene expression profile of a sample and can be used in an individualized manner without the need for normalization (14). Here, we scrutinized the prognostic significance of gene pairs for predicting outcomes in NBL.

## Materials and Methods

### Data Processing and Computational Analysis

Two public RNA-Seq data sets from the Therapeutically Applicable Research to Generate Effective Treatments (TARGET)-NBL database (https://ocg.cancer.gov/programs/target/data-matrix) and the GSE62564 data set (15) in the Gene Expression Omnibus (GEO, http://www.ncbi.nlm.nih.gov/geo) database were retrospectively analyzed to obtain clinical data. Patients who had inadequate clinical and pathologic information were excluded. Then, to uncover the practicability and accuracy of a prognostic gene pair signature for NBL, samples from the TARGET-NBL and GSE62564 cohorts were applied as training and validation cohorts, respectively. The Cancer Genome Atlas (TCGA) TARGET Genotype-Tissue Expression (GTEx) cohort (version 2016-04-12, https://toil.xenahubs.net) was used to identify the differentially expressed genes (DEGs), and fragments per kilobase of transcript per million fragments mapped (FPKM) data from the TARGET-NBL cohort (version 2018-01-07, https://ucscpublic.xenahubs.net) and reads per million (RPM) data from the GSE62564 cohort were used for the survival analysis. The background was corrected and the quintile was normalized before the *limma* package in R language (version 3.28.14) was applied for the log2-based conversion of raw data. For RNAs with multiple probes, mean expression values were calculated.

### Development and Validation of the RNA-Seq Prognostic Index (RPI)

The DEGs were selected according to *P* ≤ 0.05 and |log FC|≥1 (16, 17). The gene expression level in a specific sample or profile underwent pairwise comparison to generate a score for each gene pair (18). A gene pair score of 1 was assigned if the score of gene 1 was less than that of gene 2; otherwise, the gene pair score was 0 (18). Some gene pairs with constant values (0 or 1) in any individual data set were removed to increase reproducibility. The prognosis-related gene pairs were selected using the log-rank test to assess the association between each gene pair and patient prognosis in the training cohort. Prognostic gene pairs with a familywise error rate <0.05 were used as candidates to build the RPI. To minimize the risk of overfitting, we applied a Cox proportional hazards regression model combined with the least absolute shrinkage and selection operator (*glmnet*, version 2.0-5) (19). The penalty parameter was estimated by 10-fold cross-validation in the TARGET-NBL cohort at 1 SE beyond the minimum partial likelihood deviance (19). To divide patients into low- and high-risk groups, the optimal gene pair index cutoff value was determined by a time-dependent receiver operating characteristic (ROC) curve (*survivalROC*, version 1.0.3) (20) in the TARGET-NBL cohort. We used the nearest neighbor estimation method to estimate the ROC curve (21). The risk score was gauged by taking the score of the gene pair and the correlation coefficient into consideration, and its median was used as the cutoff to divide all subjects into two different groups: low- and high-risk groups. The *survival* package in R software was applied to perform Kaplan-Meier analysis with the log-rank test to analyze differences between the high- and low-risk groups. Heat maps were generated in Tree View, with the normalized z-score shown within each row (gene pairs). Survival prediction was assessed using a time-dependent ROC curve, and the area under the ROC curve (AUC) values were computed with the *ROCR* package (version 1.0.-7) (20, 22) to measure prognostic or predictive accuracy. Subsequently, we analyzed data in a validation cohort to assess the feasibility and reliability of this RPI model in patients with NBL. Finally, we performed a subgroup analysis in the training and validation cohorts.

### Functional Enrichment Analysis

Functional enrichment analysis was used to confirm the biological relevance of the DEGs in the training cohort using the *Moonlight* package in R software (23). The Ensemble gene IDs were converted to official gene symbols using *clusterProfiler* (version 3.3) before functional annotation and analysis. We performed the analysis in the high- and low-risk groups with the RPI.

### Development and Validation of the Clinically Adjusted RPI (RCPI)

**Figure 1:**
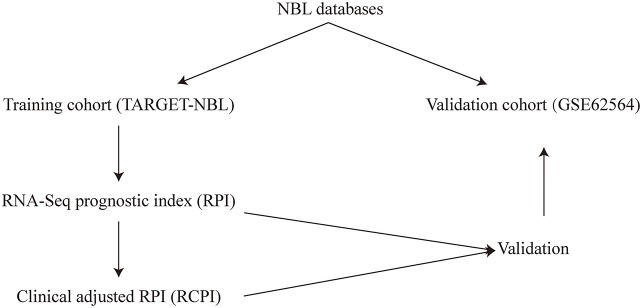
The overall workflow of this study.

The possible variables (i.e., clinicopathologic parameters) along with the RPI used to construct the RCPI were re-evaluated and then tested by the log-rank test and Cox regression analysis for the univariate and multivariate analyses, respectively. The results are presented as hazard ratios (HRs) and associated 95% confidence intervals (CIs). Based on the results of the univariate and multivariate analyses, we integrated one or more of age, stage and the RPI into a composite RCPI by applying Cox proportional hazards regression in the TARGET-NBL cohort through *My.stepwise* (version 0.10), enabling the generation of a more comparatively steady prognostic model. Different from the aforementioned method for defining the cutoff for the RPI, the cutoff value for the RCPI was estimated by medians in the corresponding cohort. The overall workflow of this study is shown in Figure 1.

### Statistical Analysis

All statistical analyses were performed using R software (version 3.5.3, 2019-03-11), in addition to the above package, *survminer* (version 0.4.6), *ggsci* (version 2.9), *tidyverse* (version 1.2.1), *cowplot* (version 1.0.0), *pheatmap* (version 1.0.12), and *ggplot2* (version 3.2.1) packages were also included. The univariate analysis of the association of clinical pathologic factors with prognosis was evaluated using the log-rank test, and the multivariate analysis was performed with the Cox proportional hazards regression model. All statistical tests were two-sided, and *P* < 0.05 was considered statistically significant.

## Results

### Identification of the Prognosis-Related DEGs

There are 1,119 clinical samples in the TARGET-NBL cohort, and 19,120 samples in the TCGA TARGET GTEx cohort had RNA-Seq gene expression data. The combination of these data revealed 10,007 DEGs. Through survival analysis combined with 190 samples from FPKM in the TARGET neuroblastoma cohort, 4,640 RNAs (2,553 RNAs are up-regulated and 2,087 RNAs are down-regulated), which were based on the DEGs, were found to be related to prognosis (see Supplementary Data Sheet 1 in the Supplementary Material for comprehensive table analysis).

### Development of the RPI

**Figure 2:**
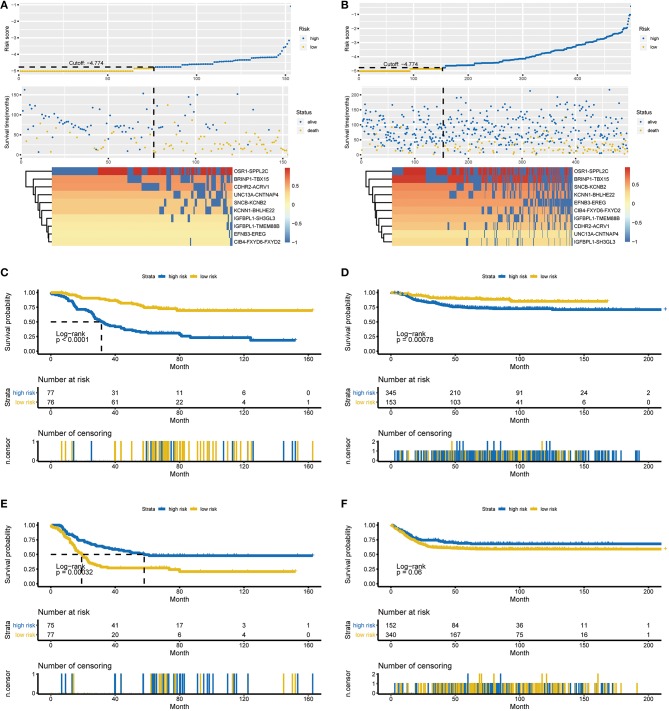
Characteristics of the RPI and Kaplan-Meier estimates for OS and EFS in the training and validation cohorts. **(A)** Characteristics of the 10-gene pair prognostic signature in the training cohort (top: the risk score of each NBL patient in the training cohort; middle: OS and survival status of patients in the training cohort; bottom: heat map of gene expression profiles of NBL patients in the training cohort). **(B)** Characteristics of the 10-gene pair prognostic signature in the validation cohort (top: the risk score of each NBL patient in the validation cohort; middle one: OS and survival status of patients in the validation cohort; bottom: heat map of gene expression profiles of NBL patients in the validation cohort). **(C)** OS in the training cohort stratified by the RPI into high- and low-risk groups with the *P*-value shown. **(D)** OS in the validation cohort stratified by the RPI into high- and low-risk groups with the *P*-value shown. **(E)** EFS in the training cohort stratified by the RPI into high- and low-risk groups with the *P*-value shown. **(F)** EFS in the validation cohort stratified by the RPI into high- and low-risk groups with the *P*-value shown. The black dotted line represents the RPI cutoff dividing patients into high- and low-risk groups, and the *P*-value was calculated using the log-rank test. RPI, RNA-Seq prognostic index; OS, overall survival, EFS, event-free survival.

A total of 651 patients with NBL (153 patients in the TARGET-NBL cohort and 498 patients in the GSE62564 cohort) were included in the present study, and the clinical and pathologic features of the patients are shown in Table 1. A complete cross-validation using the RPI was performed in the training cohort to identify a powerful prognostic signature. Through DEG and prognosis-related RNA analysis, 81 RNAs were used as candidates to build gene pairs. The strong association of the 31 RNAs (*P* < 0.01) with OS was assessed in the TARGET-NBL cohort, resulting in 10 prognostic gene pairs (Table 2). Through this procedure, the RPI was determined by using L1-penalized Cox proportional hazards regression, and the usefulness of outcome prediction was assessed for the first time. The 10 gene pairs of the RPI were selected at a significantly higher frequency than were those by different randomizations. On the basis of the time-dependent ROC curve analysis, the optimal cutoff value that could be used for the RPI to stratify patients into high or low risk group was determined to be −4.774 (see Figure 2A). The regression coefficients from this model were used to construct the RPI, and a threshold was chosen at the median manually. The RPI was calculated according to the gene pairs, RPI = (−0.250693546 ^*^ CDHR2-ACRV1) + (−0.410999623 ^*^ SNCB-KCNB2) + (−0.168095779 ^*^ BRINP1-TBX15) + (−0.384018203 ^*^ KCNN1-BHLHE22) + (−1.48200304 ^*^ EFNB3-EREG) + (−0.429605222 ^*^ UNC13A-CNTNAP4) + (−0.424862085 ^*^ IGFBPL1-SH3GL3) + (−0.878692179 ^*^ IGFBPL1-TMEM88B) + (0.002590887 ^*^ OSR1-SPPL2C) + (−0.595746694 ^*^ CIB4-FXYD6-FXYD2).

**Table 1:** Clinical and pathologic features of patients in the TARGET-NBL and GSE62564 cohorts.

	**TARGET-NBL (*N* = 153)**	**GSE62564 (*N* = 498)**	**Total (*N* = 651)**
**Gender**
Male	89 (58.17%)	287 (57.63%)	376 (57.76%)
Female	64 (41.83%)	211 (42.37%)	275 (42.24%)
**Age at diagnosis in days**
≥18 months	124 (81.05%)	193 (38.76%)	317 (48.69%)
<18 months	29 (18.95%)	305 (61.24%)	334 (51.31%)
**MYCN status**
Amplified	33 (21.57%)	92 (18.47%)	125 (19.20%)
Not Amplified	119 (77.78%)	401 (80.52%)	520 (79.88%)
Unknown	1 (0.65%)	5 (1.01%)	6 (0.92%)
**Stage**
4	126 (82.35%)	183 (36.75%)	309 (47.47%)
1, 2, 3, 4S	27 (17.65%)	314 (63.05%)	341 (52.38%)
Unknown	–	1 (0.20%)	1 (0.15%)

**Table 2:** Details of 10 gene pairs.

**Gene pair**	**Gene 1**	**Full name**	**Gene 2**	**Full name**	**Coefficient**
ENSG00000074276.ENSG00000134940	CDHR2	Cadherin related family member 2	ACRV1	Acrosomal vesicle protein 1	−0.2506935
ENSG00000074317.ENSG00000182674	SNCB	Synuclein beta	KCNB2	Potassium voltage-gated channel subfamily B member 2	−0.4109996
ENSG00000078725.ENSG00000092607	BRINP1	BMP/Retinoic acid inducible neural specific 1	TBX15	T-Box 15	−0.1680958
ENSG00000105642.ENSG00000180828	KCNN1	Potassium calcium-activated channel subfamily N member 1	BHLHE22	Basic helix-loop-helix family member E22	−0.3840182
ENSG00000108947.ENSG00000124882	EFNB3	Ephrin B3	EREG	Epiregulin	−1.482003
ENSG00000130477.ENSG00000152910	UNC13A	Unc-13 homolog A	CNTNAP4	Contactin associated protein like 4	−0.4296052
ENSG00000137142.ENSG00000140600	IGFBPL1	Insulin like growth factor binding protein like 1	SH3GL3	SH3 domain containing GRB2 like 3, endophilin A3	−0.4248621
ENSG00000137142.ENSG00000205116	IGFBPL1	Insulin like growth factor binding protein like 1	TMEM88B	Transmembrane protein 88B	−0.8786922
ENSG00000143867.ENSG00000185294	OSR1	Odd-skipped related transcription factor 1	SPPL2C	Signal peptide peptidase like 2C	0.00259089
ENSG00000157884.ENSG00000255245	CIB4	Calcium and integrin binding family member 4	FXYD6-FXYD2	FXYD6-FXYD2 readthrough	−0.5957467

All 153 patients in the training cohort were segregated into the low-risk group (*n* = 76) and the high-risk group (*n* = 77), and the low-risk group exhibited significantly better overall survival (OS) than the high-risk group according to the RPI cutoff point (*P* < 0.0001, see Figure 2C). For the low-risk group, the median OS was not reached, whereas the median OS was 31.5 months (95% CI: 26.7–45.9) for the high-risk group. The HR for progression in the low- vs. high-risk group was 0.24 (95% CI: 0.15–0.41, *P* < 0.001). Regarding event-free survival (EFS), all 152 patients in the training cohort (1 patient was removed due to incomplete information) were similarly segregated into the low- and high-risk groups, and a low RPI was correlated with significantly favorable EFS in the TARGET-NBL cohort (*P* = 0.00032, see Figure 2E). The median EFS was 58.0 months (95% CI: 36.3–NA) and 19.2 months (95% CI: 15.4–23.5) for the high- and low-risk groups, respectively (HR = 2.11, 95% CI: 1.39–3.21, *P* = 0.000435). The subgroup analysis showed that our RPI could well divide patients into different risk groups and correlated with prognosis (*P* < 0.01 for all subgroups except for age <18 months and stage 1, 2, 3, and 4S subgroups in the validation cohort, see Figure S1 in the Supplementary Material for the comprehensive figure analysis).

### Validation of the RPI

To examine the robust and realistic application of the RPI, the performance of the RPI was validated in the validation cohort (see Figure 2B). The developed model could actively predict OS and EFS in patients with NBL in the validation cohort. The RPI significantly stratified patients into low- and high-risk groups in terms of OS; more specifically, all 498 patients were segregated into the low-risk group (*n* = 153) and the high-risk group (*n* = 345) and showed significantly different OS rates (*P* = 0.00078) according to the same risk score cutoff point (−4.774) acquired from the training cohort (see Figure 2D). The median OS was not reached in either the low- or high-risk group, and the HR for progression in the low- vs. high-risk group was 0.43 (95% CI: 0.26–0.71, *P* = 0.005). Concerning EFS, all 492 patients in the training cohort (6 patients were removed due to incomplete information) were similarly divided into the low- and high-risk groups, and a low RPI tended to favor favorable EFS (*P* = 0.06, see Figure 2F). The median EFS was not reached in either the low- or high-risk group, and the HR for progression in the low- vs. high-risk group was 1.38 (95% CI: 0.99–1.93, *P* = 0.0608). Overall, the RPI appears to independently estimate OS and EFS in patients with NBL well. However, the subgroup analysis showed that the RPI did not perform well (see Figure S2 in the Supplementary Material for the comprehensive figure analysis).

### Performance Comparison by Time-Dependent ROC Curve Analysis

**Figure 3:**
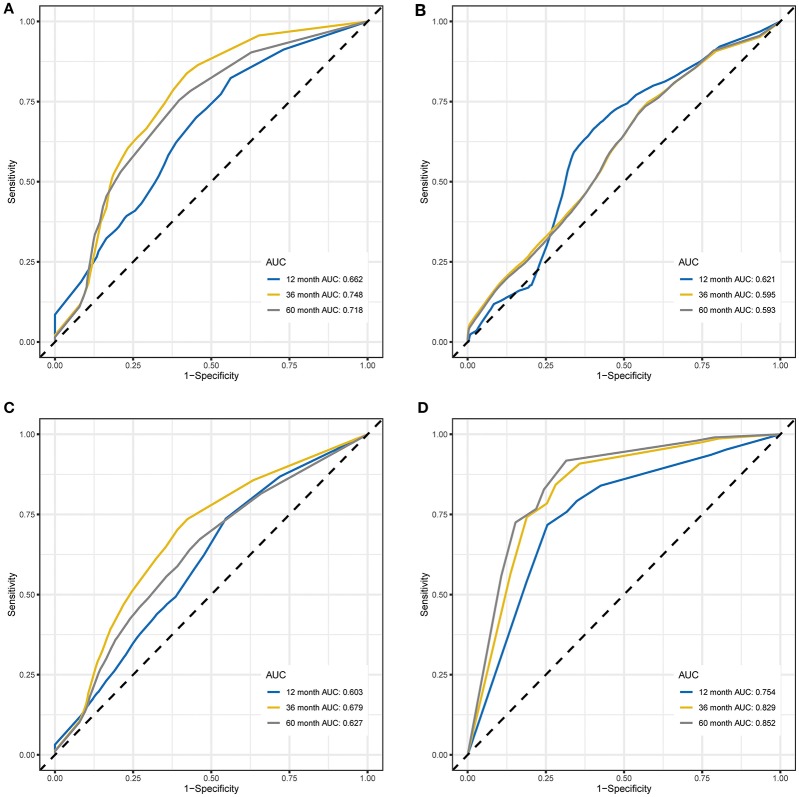
Time-dependent ROC curves for OS and EFS predicted with the RPI in the training and validation cohorts. **(A)** Time-dependent ROC curves for OS predicted with the RPI in the training cohort. **(B)** Time-dependent ROC curves for OS predicted with the RPI in the validation cohort. **(C)** Time-dependent ROC curves for EFS predicted with the RPI in the training cohort. **(D)** Time-dependent ROC curves for EFS predicted with the RPI in the validation cohort. AUC values at 12, 36, and 60 months were used to assess the prognostic accuracy. ROC, receiver operating characteristic; OS, overall survival; EFS, event-free survival; AUC, area under the ROC curve.

Time-dependent ROC curve analysis was performed to compare the sensitivity and specificity of the prediction of OS and EFS with the RPI in the training and validation cohorts. The AUC value was obtained from ROC curve analysis. Regarding OS, in the training and validation cohorts, the RPI reached 12 month AUC values of 0.662 and 0.621, 36 month AUC values of 0.748 and 0.595, and 60 month AUC values of 0.718 and 0.593, respectively (see Figures 3A,B), demonstrating that the predictive power of the RPI was credible in both the training and validation cohorts. Upon calculating the AUC of EFS, we obtained the same results (see Figures 3C,D).

### Correlated Functional Enrichment Analysis

**Figure 4:**
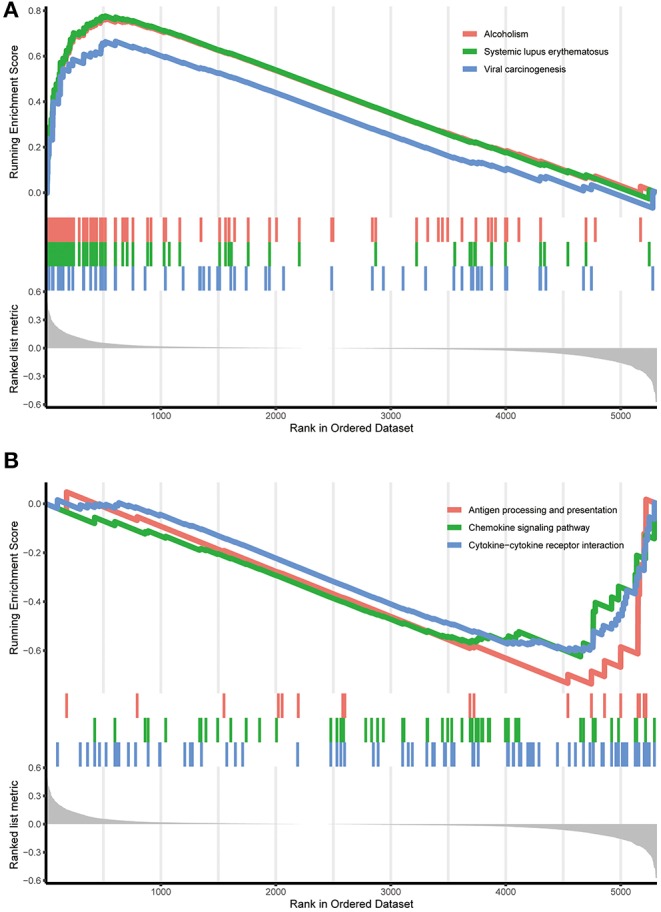
Functional enrichment of the DEGs in the training cohort. **(A)** Functional enrichment in the high-risk group identified by the RPI. **(B)** Functional enrichment in the low-risk group identified by the RPI. RPI, RNA-Seq prognostic index.

To scrutinize the functional implications of the DEGs in NBL initiation and progression, bioinformatics analysis was performed. We found that expression alterations in these genes could activate alcoholism, systemic lupus erythematosus and viral carcinogenesis in the high-risk group, whereas antigen processing and presentation, the chemokine signaling pathway and the cytokine-cytokine receptor interaction were activated in the low-risk group (see Figure 4).

### Development and Validation of the RCPI

**Figure 5:**
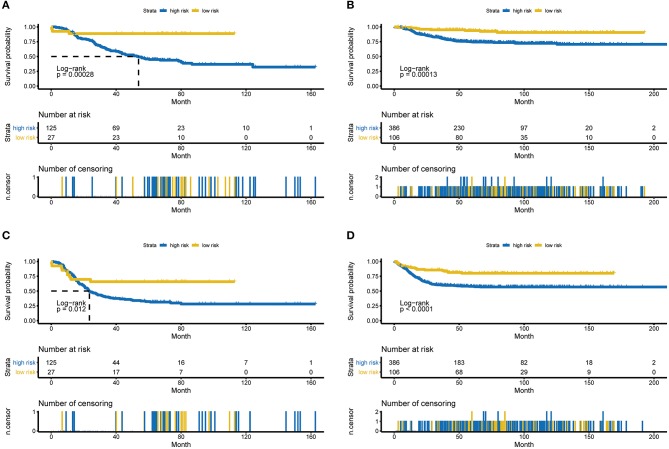
Kaplan-Meier estimates for OS and EFS predicted with the RCPI in the training and validation cohorts. **(A)** OS in the training cohort stratified by the RCPI into high- and low-risk groups with the *P*-value shown. **(B)** OS in the validation cohort stratified by the RCPI into high- and low-risk groups with the *P*-value shown. **(C)** EFS in the training cohort stratified by the RCPI into high- and low-risk groups with the *P*-value shown. **(D)** EFS in the validation cohort stratified by the RCPI into high- and low-risk groups with the *P*-value shown. The *P*-value was calculated using the log-rank test. OS, overall survival; EFS, event-free survival; RCPI, clinically adjusted RNA-Seq prognostic index.

**Figure 6:**
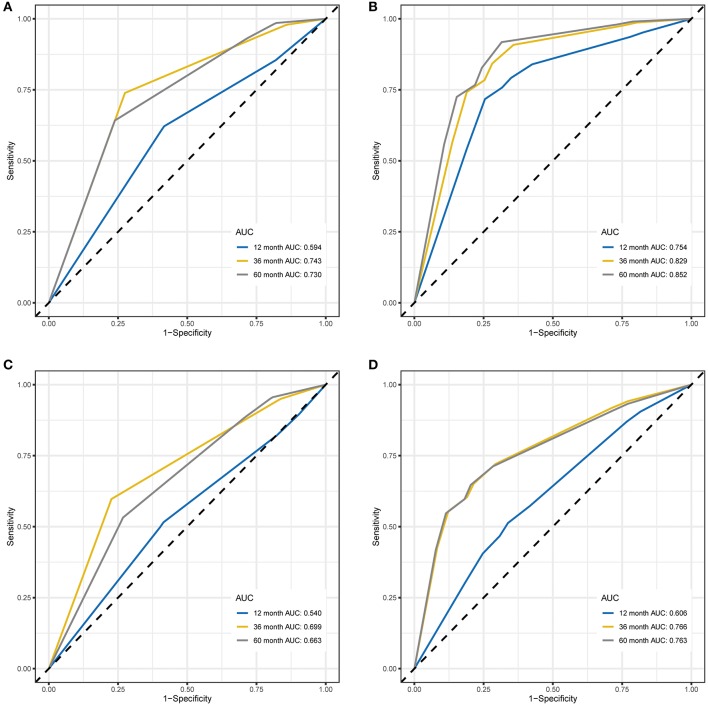
Time-dependent ROC curves for OS and EFS predicted with the RCPI in the training and validation cohorts. **(A)** Time-dependent ROC curves for OS predicted with the RCPI in the training cohort. **(B)** Time-dependent ROC curves for OS predicted with the RCPI in the validation cohort. **(C)** Time-dependent ROC curves for EFS predicted with the RCPI in the training cohort. **(D)** Time-dependent ROC curves for EFS predicted with the RCPI in the validation cohort. AUC values at 12, 36, and 60 months were used to assess the prognostic accuracy. ROC, receiver operating characteristic; OS, overall survival; EFS, event-free survival; RCPI, clinically adjusted RNA-Seq prognostic index; AUC, area under the ROC curve.

Several of the clinicopathological features mentioned earlier were considered possible predictors. Univariate and multivariate analyses were first performed to further investigate which parameters could be used to better estimate the results. As shown in Table 3, in the univariate analysis, prognosis was correlated with age, stage and the RPI in the training cohort and with age, stage and MYCN in the validation cohort (all *P* < 0.05). The multivariate analysis confirmed that the RPI independently predicted prognosis in the training cohort and age, stage, and MYCN in the validation cohort (all *P* < 0.05). In short, we propose that stage and the RPI are complementary. To further improve the accuracy, we ultimately combined stage and the RPI to fit a preferable model in the training cohort and subsequently validated the model in the validation cohort [RCPI = (stage ^*^ 1.8445) + (RPI ^*^ 1.0563)]. The optimal cutoff value used to stratify patients was determined based on the median value in the corresponding cohort. Then, we applied the RCPI in the training and validation cohorts to test its differentiation, accuracy and specificity to predict OS and EFS. We found that the RCPI could well divide patients into high- and low-risk groups that and the low-risk group had a better prognosis (all *P* < 0.05, see Figure 5). As shown in Figure 6, the sensitivity and specificity of the RCPI increased over time.

**Table 3:** Univariate and multivariate Cox regression analyses of clinicopathological factors.

**Datasets**	**Variables**	**Univariate analysis**		**Multivariate analysis**	
		**HR (95% CI)**	***P***	**HR (95% CI)**	***P***
TARGET-NBL	Gender	1.05 (0.67–1.67)	0.822	0.92 (0.57–1.50)	0.748
	Age	4.04 (1.63–10.04)	**0.003**	1.15 (0.29–4.54)	0.841
	Stage	6.46 (2.03–20.52)	**0.002**	5.44 (0.96–30.65)	0.055
	MYCN	1.39 (0.82–2.36)	0.226	1.08 (0.63–1.86)	0.771
	RPI	2.91 (1.79–4.74)	**<0.001**	2.91 (1.76–4.81)	**<0.001**
GSE62564	Gender	0.82 (0.55–1.20)	0.299	0.74 (0.50–1.10)	0.138
	Age	8.58 (5.26–14)	**<0.001**	3.59 (2.07–6.23)	**<0.001**
	Stage	8.54 (5.36–13.6)	**<0.001**	3.37 (1.99–5.72)	**<0.001**
	MYCN	7.78 (5.26–11.53)	**<0.001**	3.39 (2.24–5.15)	**<0.001**
	RPI	1.53 (0.97–2.42)	0.067	1.03 (0.64–1.65)	**<0.001**

*Bold values indicate the statistical difference*.

## Discussion

In the last few decades, significant breakthroughs have deepened our understanding of the tumorigenesis and development of NBL. NBL most frequently arises from neuronal cells that fail to differentiate into the adrenal medulla but can also develop in the neck, chest, abdomen, or spine (24), and different sources have different genomic profiles (25). Based on its molecular and clinical features, patients are classified into four different risk groups and have different prognoses (26). However, the clinical prognosis of patients with NBL remains highly diverse (25, 27). Hence, it is necessary to determine the biological characteristics of NBL patients (28). Some genetic susceptibility factors are strongly associated with NBL. Germline mutations in ALK explain most hereditary NBLs (8, 9). Germline mutations in PHOX2B (13) or KIF1Bβ (29) have also been implicated in familial NBL. MYCN has been found to be amplified in NBL (7). NBL has been linked to copy number variations within NBPF10 (30) and single nucleotide polymorphism variations within FLJ22536 (31) and BARD1 (32), as well as duplicated segments of LMO1 (10, 11). DOT1L (33), RBPJ and SNW1 (34) are upregulated in NBL and associated with unfavorable patient outcomes. Patients with advanced-stage NBL who express high levels of TNIP1 and N4BP1 exhibit poor OS (35). High levels of RUNX3 result in a preferable prognosis in patients with NBL (36). Generally, these findings indicate that common DNA variations influence NBL and promote the development of a putative genetic model for this disease (37).

Here, we developed and proposed an RCPI that is significantly associated with outcome prediction, making it a favorable and practical tool for risk classification in patients with NBL. The individualized RCPI is not described in the International Neuroblastoma Risk Group Staging System (INRGSS), which was created specifically to constitute one of seven prognostic factors in the International Neuroblastoma Risk Group (INRG) pretreatment classification system (26, 38). The high prognostic categorization performance of the RCPI is assuredly due to our idiographic reanalysis strategy. To identify reliable prognostic biomarkers of NBL, we utilized new methods of multi-gene analysis. First, only RNA-Seq data were included, which resulted in the greatest difference observed among all previous studies (39, 40); thus, our study did not overlook genes that appeared only in the array. Furthermore, we found that data in the TARGET-NBL cohort are profiled as FPKM and that data in the GSE62564 cohort are profiled as RPM. Due to this difference, the gene pairs were homogenized; therefore, the score was calculated based entirely on the gene expression profile of a sample and was used in an individualized manner without the need for normalization (14). Third, our results indicate that the RPI can also be applied to the subgroups of sex and MYCN in the TARGET-NBL cohort. Moreover, because of the RPI, we not only obtained many clinicopathological features from the univariate and multivariate analyses that are strongly and significantly correlated with prognosis and deserve further study but also further used the same powerful algorithm to generate the RCPI, a preferable model combining clinicopathological features with the RPI. With the addition of clinicopathological features, the prediction of OS and EFS by the RCPI for NBL patients is quite encouraging; moreover, the longer the prediction time is, the more accurate the model is.

Furthermore, functional enrichment analysis indicated that these DEGs may be involved in certain events that are associated with NBL. Studies have shown that alcohol use during pregnancy increases the risk of NBL (41). According to the report, there is a unique coincidence of neonatal lupus and NBL (42). In addition, antigen processing and presentation are also involved in the treatment of NBL (43, 44). The chemokine signaling pathway (45, 46) and the cytokine-cytokine receptor interaction (47) also play a role in the occurrence and development of NBL.

There are some limitations to this study, although the RCPI is robust. First, the public RNA-Seq data sets included in this analysis were profiled from different platforms: TARGET-NBL was profiled from FPKM, and GSE62564 was profiled from RPM. Despite homogenization, bias still exists, which may lead to poor extrapolation of the findings. Second, limited by the clinical information included in the data sets, we were not able to single out the high-risk group, as in a previous study (39). This may also be the reason why we did not obtain satisfactory results in the GSE62564 cohort when conducting the subgroup analysis (i.e., because of the asymmetry of clinical data between the GSE62564 and TARGET-NBL cohorts). Third, although different databases were used as the training and validation cohorts, the clinical sample size of each cohort included in this study was still relatively small. Therefore, it is still necessary to study large samples in future studies. Finally, this was a data mining study; therefore, multicenter, well-designed, prospective studies are needed to validate these findings. Despite these drawbacks, independent confirmation and similarities between findings from the training and validation cohorts provide a high level of confidence in the overall analysis.

The RCPI may be the first prognostic tool in NBL that combines clinicopathologic characteristics with laboratory test indicators. Furthermore, it was proven that the prognostic power of the RCPI, an optimal signature with outcome prediction, was acceptable. As such, our RCPI can serve as a personalized, single-sample estimate of survival in NBL patients and may be promptly incorporated into clinical utility. More importantly, this useful strategy for the vigorous selection of prognostic markers has vast application potential in other diseases.

## Conclusions

The proposed novel RCPI is a promising prognostic signature in NBL. Prospective and large-sample studies are needed to further validate its penetrating precision for estimating prognoses and to verify its use in clinical practice for the personalized therapy management of NBL.

## Data Availability Statement

RNA-Seq datasets were acquired from the Therapeutically Applicable Research to Generate Effective Treatments (TARGET)-NBL database (https://ocg.cancer.gov/programs/target/data-matrix) and the GSE62564 dataset in the Gene Expression Omnibus (GEO, http://www.ncbi.nlm.nih.gov/geo) database.

## Author Contributions

J-GZ, S-HJ, HM, and UG conceived, designed, and planned the study. BL and J-GZ analyzed the data. G-BD and LC acquired the data. J-GZ, S-HJ, BL, H-LL, and HM helped interpret the results. J-GZ and S-HJ provided study materials or patients. J-GZ, BL, and HM drafted the manuscript. All authors revised and reviewed this work and gave their final approval of the submitted manuscript.

### Conflict of Interest

The authors declare that the research was conducted in the absence of any commercial or financial relationships that could be construed as a potential conflict of interest.

## Supplementary Material

Supplementary Data Sheet 1 The details of 4,640 prognosis-related differentially expressed RNAs.

Figure S1 The subgroup analysis of RPI in the training cohort. **(A)** The gender subgroup analysis of RPI in the training cohort (The left one is male and the right one is female). **(B)** The age subgroup analysis of RPI in the training cohort (The left one is ≥ 18 months and the right one is < 18 months). **(C)** The stage subgroup analysis of RPI in the training cohort (The left one is stage 4 and the right one is stage 1 ~ 3 and 4S). **(D)** The MYCN subgroup analysis of RPI in the training cohort (The left one is amplified and the right one is not amplified).

Figure S2 The subgroup analysis of RPI in the validation cohort. **(A)** The gender subgroup analysis of RPI in the training cohort (The left one is male and the right one is female). **(B)** The age subgroup analysis of RPI in the training cohort (The left one is ≥ 18 months and the right one is < 18 months). **(C)** The stage subgroup analysis of RPI in the training cohort (The left one is stage 4 and the right one is stage 1 ~ 3 and 4S). **(D)** The MYCN subgroup analysis of RPI in the training cohort (The left one is amplified and the right one is not amplified).

## Abbreviations

Age: age at diagnosis in days
AUC: area under the receiver operating characteristic curve
CI: confidence interval
DEG: differentially expressed gene
EFS: event-free survival
FPKM: fragments per kilobase of transcript per million fragments mapped
GTEx: Genotype-Tissue Expression
HR: hazard ratio
INRG: International Neuroblastoma Risk Group
INRGSS: International Neuroblastoma Risk Group Staging System
NBL: neuroblastoma
OS: overall survival
RCPI: clinically adjusted RNA-Seq prognostic index
ROC: receiver operating characteristic
RPI: RNA-Seq prognostic index
RPM: reads per million
TARGET: Therapeutically Applicable Research to Generate Effective Treatments
TCGA: The Cancer Genome Atlas.

## References

[1] Maris JM Hogarty MD Bagatell R Cohn SL. Neuroblastoma. Lancet. (2007) 369:2106–20. 10.1016/S0140-6736(07)60983-0 17586306

[2] Zaman S Chobrutskiy BI Blanck G. MAPT (Tau) expression is a biomarker for an increased rate of survival in pediatric neuroblastoma. Cell Cycle. (2018) 17:2474–83. 10.1080/15384101.2018.1542898 30394813 PMC6342068

[3] Whittle SB Smith V Doherty E Zhao S McCarty S Zage PE. Overview and recent advances in the treatment of neuroblastoma. Expert Rev Anticancer Ther. (2017) 17:369–86. 10.1080/14737140.2017.1285230 28142287

[4] Lucas JTJ McCarville MB Cooper DA Doubrovin M Wakefield D Santiago T. Implications of image-defined risk factors and primary-site response on local control and radiation treatment delivery in the management of high-risk neuroblastoma: is there a role for de-escalation of adjuvant primary-site radiation therapy? Int J Radiat Oncol Biol Phys. (2019) 103:869–77. 10.1016/j.ijrobp.2018.11.041 30496881 PMC8810202

[5] De Preter K Vermeulen J Brors B Delattre O Eggert A Fischer M. Accurate outcome prediction in neuroblastoma across independent data sets using a multigene signature. Clin Cancer Res. (2010) 16:1532–41. 10.1158/1078-0432.CCR-09-2607 20179214

[6] Borriello L Seeger RC Asgharzadeh S DeClerck YA. More than the genes, the tumor microenvironment in neuroblastoma. Cancer Lett. (2016) 380:304–14. 10.1016/j.canlet.2015.11.017 26597947 PMC5558454

[7] Brodeur GM Seeger RC Schwab M Varmus HE Bishop JM. Amplification of N-myc in untreated human neuroblastomas correlates with advanced disease stage. Science. (1984) 224:1121–4. 10.1126/science.6719137 6719137

[8] Mosse YP Laudenslager M Longo L Cole KA Wood A Attiyeh EF. Identification of ALK as a major familial neuroblastoma predisposition gene. Nature. (2008) 455:930–5. 10.1038/nature07261 18724359 PMC2672043

[9] Pugh TJ Morozova O Attiyeh EF Asgharzadeh S Wei JS Auclair D. The genetic landscape of high-risk neuroblastoma. Nat Genet. (2013) 45:279–84. 10.1038/ng.2529 23334666 PMC3682833

[10] Wang K Diskin SJ Zhang H Attiyeh EF Winter C Hou C. Integrative genomics identifies LMO1 as a neuroblastoma oncogene. Nature. (2011) 469:216–20. 10.1038/nature09609 21124317 PMC3320515

[11] Oldridge DA Wood AC Weichert-Leahey N Crimmins I Sussman R Winter C. Genetic predisposition to neuroblastoma mediated by a LMO1 super-enhancer polymorphism. Nature. (2015) 528:418–21. 10.1038/nature15540 26560027 PMC4775078

[12] Peng P-L Zhou X-Y Yi G-D Chen P-F Wang F Dong W-G. Identification of a novel gene pairs signature in the prognosis of gastric cancer. Cancer Med. (2018) 7:344–50. 10.1002/cam4.1303 29282891 PMC5806102

[13] Boeva V Louis-Brennetot C Peltier A Durand S Pierre-Eugene C Raynal V. Heterogeneity of neuroblastoma cell identity defined by transcriptional circuitries. Nat Genet. (2017) 49:1408–13. 10.1038/ng.3921 28740262

[14] Li B Cui Y Diehn M Li R. Development and validation of an individualized immune prognostic signature in early-stage nonsquamous non-small cell lung cancer. JAMA Oncol. (2017) 3:1529–37. 10.1001/jamaoncol.2017.1609 28687838 PMC5710196

[15] Consortium SM-I A comprehensive assessment of RNA-seq accuracy, reproducibility and information content by the sequencing quality control consortium. Nat Biotechnol. (2014) 32:903–14. 10.1038/nbt.2957 25150838 PMC4321899

[16] Robertson DS Wildenhain J Javanmard A Karp NA. OnlineFDR: an R package to control the false discovery rate for growing data repositories. Bioinformatics. (2019) 35:4196–9. 10.1093/bioinformatics/btz191 30873526 PMC6792083

[17] Pawitan Y Michiels S Koscielny S Gusnanto A Ploner A. False discovery rate, sensitivity and sample size for microarray studies. Bioinformatics. (2005) 21:3017–24. 10.1093/bioinformatics/bti448 15840707

[18] Kim S Lin C-W Tseng GC. MetaKTSP: a meta-analytic top scoring pair method for robust cross-study validation of omics prediction analysis. Bioinformatics. (2016) 32:1966–73. 10.1093/bioinformatics/btw115 27153719 PMC6280887

[19] Simon N Friedman J Hastie T Tibshirani R. Regularization paths for Cox's proportional hazards model via coordinate descent. J Stat Softw. (2011) 39:1–13. 10.18637/jss.v039.i05 27065756 PMC4824408

[20] Heagerty PJ Lumley T Pepe MS. Time-dependent ROC curves for censored survival data and a diagnostic marker. Biometrics. (2000) 56:337–44. 10.1111/j.0006-341X.2000.00337.x 10877287

[21] Akritas MG Nearest neighbor estimation of a bivariate distribution under random censoring. Ann Statist. (1994) 22:1299–327. 10.1214/aos/1176325630

[22] Paul B Jean-François D Hélène JG Estimating and comparing time-dependent areas under receiver operating characteristic curves for censored event times with competing risks. Statist Med. (2013) 32:5381–97. 10.1002/sim.5958 24027076

[23] Colaprico A Olsen C Cava C Terkelsen T Silva TC Olsen A Moonlight: a tool for biological interpretation and driver genes discovery. (2018) bioRxiv 265322. 10.1101/265322

[24] Nakagawara A Izumi H Li Y Muramori K Inada H Nishi M. Neuroblastoma. Jpn J Clin Oncol. (2018) 48:214–41. 10.1093/jjco/hyx176 29378002

[25] Oldridge DA Truong B Russ D DuBois SG Vaksman Z Mosse YP. Differences in genomic profiles and outcomes between thoracic and adrenal neuroblastoma. J Natl Cancer Inst. (2019) 111:1192–201. 10.1093/jnci/djz027 30793172 PMC6855946

[26] Cohn SL Pearson ADJ London WB Monclair T Ambros PF Brodeur GM. The International Neuroblastoma Risk Group (INRG) classification system: an INRG Task Force report. J Clin Oncol. (2009) 27:289–97. 10.1200/JCO.2008.16.6785 19047291 PMC2650388

[27] Haupt R Garaventa A Gambini C Parodi S Cangemi G Casale F. Improved survival of children with neuroblastoma between 1979 and 2005: a report of the Italian Neuroblastoma Registry. J Clin Oncol. (2010) 28:2331–8. 10.1200/JCO.2009.24.8351 20351331

[28] Davidoff AM. Neuroblastoma. Semin Pediatr Surg. (2012) 21:2–14. 10.1053/j.sempedsurg.2011.10.009 22248965 PMC3261589

[29] Fell SM Li S Wallis K Kock A Surova O Rraklli V. Neuroblast differentiation during development and in neuroblastoma requires KIF1Bbeta-mediated transport of TRKA. Genes Develop. (2017) 31:1036–53. 10.1101/gad.297077.117 28637693 PMC5495120

[30] Diskin SJ Hou C Glessner JT Attiyeh EF Laudenslager M Bosse K. Copy number variation at 1q21.1 associated with neuroblastoma. Nature. (2009) 459:987–91. 10.1038/nature08035 19536264 PMC2755253

[31] Maris JM Mosse YP Bradfield JP Hou C Monni S Scott RH. Chromosome 6p22 locus associated with clinically aggressive neuroblastoma. N Engl J Med. (2008) 358:2585–93. 10.1056/NEJMoa0708698 18463370 PMC2742373

[32] Capasso M Devoto M Hou C Asgharzadeh S Glessner JT Attiyeh EF. Common variations in BARD1 influence susceptibility to high-risk neuroblastoma. Nat Genet. (2009) 41:718–23. 10.1038/ng.374 19412175 PMC2753610

[33] Wong M Tee AEL Milazzo G Bell JL Poulos RC Atmadibrata B. The Histone methyltransferase DOT1L promotes neuroblastoma by regulating gene transcription. Cancer Res. (2017) 77:2522–33. 10.1158/0008-5472.CAN-16-1663 28209620

[34] Hong M He J Li S SNW1 regulates Notch signaling in neuroblastoma through interacting with RBPJ.Biochem Biophys Res Commun. (2019) 509:869–76. 10.1016/j.bbrc.2019.01.036 30642633

[35] Spel L Nieuwenhuis J Haarsma R Stickel E Bleijerveld OB Altelaar M. Nedd4-binding protein 1 and TNFAIP3-interacting protein 1 control MHC-1 display in neuroblastoma. Cancer Res. (2018) 78:6621–31. 10.1158/0008-5472.CAN-18-0545 30213788

[36] Yu F Gao W Yokochi T Suenaga Y Ando K Ohira M. RUNX3 interacts with MYCN and facilitates protein degradation in neuroblastoma. Oncogene. (2014) 33:2601–9. 10.1038/onc.2013.221 23851507

[37] Maris JM. Recent advances in neuroblastoma. N Engl J Med. (2010) 362:2202–11. 10.1056/NEJMra0804577 20558371 PMC3306838

[38] Monclair T Brodeur GM Ambros PF Brisse HJ Cecchetto G Holmes K. The International Neuroblastoma Risk Group (INRG) staging system: an INRG Task Force report. J Clin Oncol. (2009) 27:298–303. 10.1200/JCO.2008.16.6876 19047290 PMC2650389

[39] Sahu D Ho S-Y Juan H-F Huang H-C. High-risk, expression-based prognostic long noncoding RNA signature in neuroblastoma. JNCI Cancer Spectr. (2018) 2:pky015. 10.1093/jncics/pky015 31360848 PMC6649748

[40] Zhong X Liu Y Liu H Zhang Y Wang L Zhang H. Identification of potential prognostic genes for neuroblastoma. Front Genet. (2018) 9:589. 10.3389/fgene.2018.00589 30555514 PMC6282001

[41] Heck JE Ritz B Hung RJ Hashibe M Boffetta P. The epidemiology of neuroblastoma: a review. Paediatr Perinat Epidemiol. (2009) 23:125–43. 10.1111/j.1365-3016.2008.00983.x 19159399

[42] Sharawat IK Saini AG Kasinathan A Mandava SS Sankhyan N. Neuroblastoma, opsoclonus-myoclonus ataxia syndrome and neonatal lupus with congenital heart block: is there an association? Lupus. (2018) 27:2298–9. 10.1177/0961203318804339 30282557

[43] Pastor ER Mousa SA. Current management of neuroblastoma and future direction. Crit Rev Oncol Hematol. (2019) 138:38–43. 10.1016/j.critrevonc.2019.03.013 31092383

[44] Peinemann F van Dalen EC Enk H Tytgat GA. Anti-GD2 antibody-containing immunotherapy postconsolidation therapy for people with high-risk neuroblastoma treated with autologous haematopoietic stem cell transplantation. Cochrane Database Syst Rev. (2019) 4:Cd012442. 10.1002/14651858.CD012442.pub2 31016728 PMC6479178

[45] Dedoni S Campbell LA Harvey BK Avdoshina V. The orphan G-protein-coupled receptor 75 signaling is activated by the chemokine CCL5. J Neurochem. (2018) 146:526–39. 10.1111/jnc.14463 29772059 PMC6198807

[46] Zhi Y Lu H Duan Y Sun W Guan G Dong Q. Involvement of the nuclear factor-kappaB signaling pathway in the regulation of CXC chemokine receptor-4 expression in neuroblastoma cells induced by tumor necrosis factor-alpha. Int J Mol Med. (2015) 35:349–57. 10.3892/ijmm.2014.2032 25503960 PMC4292717

[47] Lelievre E Plun-Favreau H Chevalier S Froger J Guillet C Elson GC. Signaling pathways recruited by the cardiotrophin-like cytokine/cytokine-like factor-1 composite cytokine: specific requirement of the membrane-bound form of ciliary neurotrophic factor receptor alpha component. J Biol Chem. (2001) 276:22476–84. 10.1074/jbc.M101681200 11294841

