# Supplementary figures and images for: Development and Validation of an RNA-Seq-Based Prognostic Signature in Neuroblastoma
Source: Front Oncol. 2019 Dec 4;9:1361. doi: 10.3389/fonc.2019.01361 (PMC6904333; doi:10.3389/fonc.2019.01361)

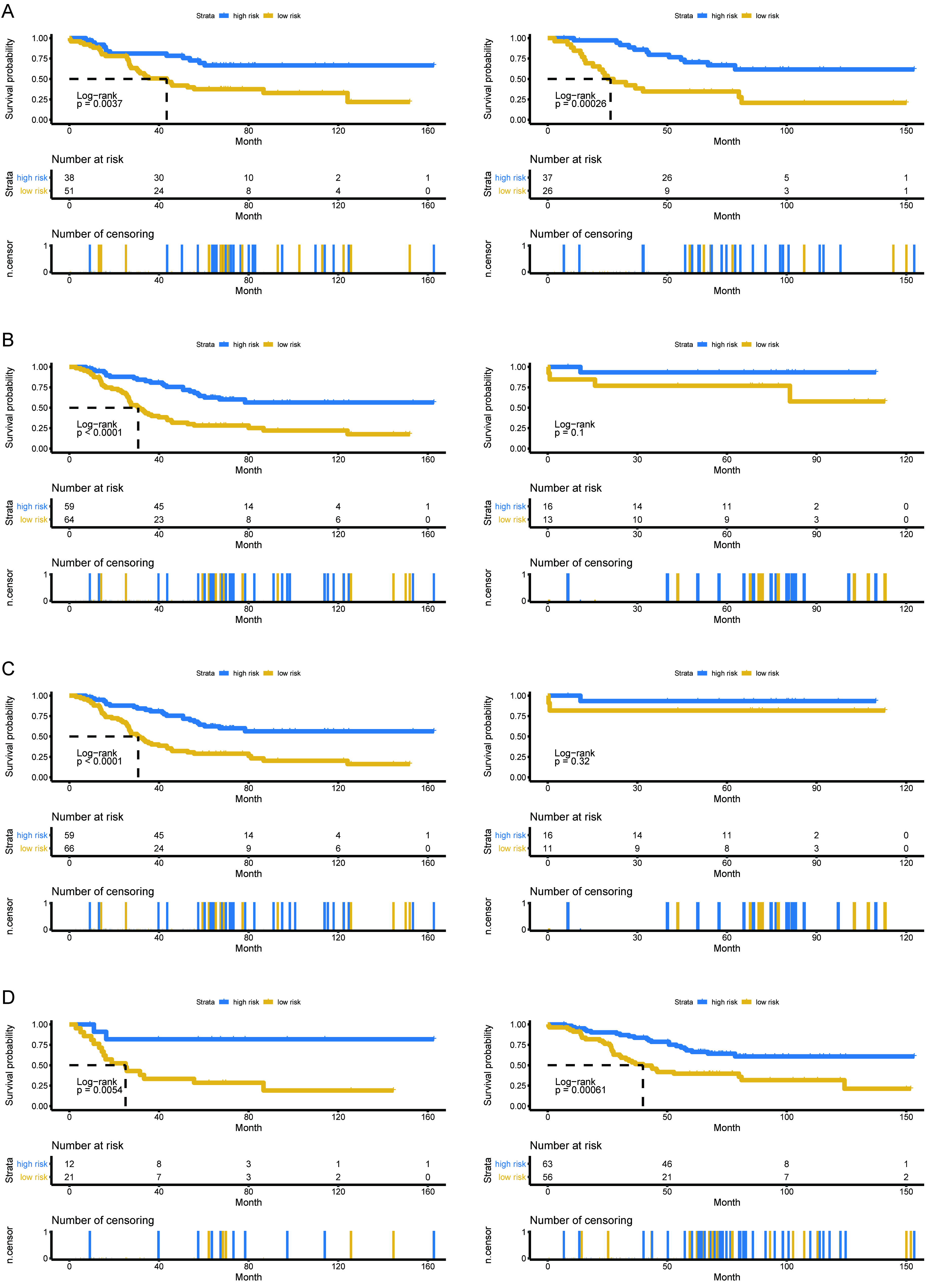

Supplement: Figure S1 — The subgroup analysis of RPI in the training cohort. (A) The gender subgroup analysis of RPI in the training cohort (The left one is male and the right one is female). (B) The age subgroup analysis of RPI in the training cohort (The left one is ≥ 18 months and the right one is < 18 months). (C) The stage subgroup analysis of RPI in the training cohort (The left one is stage 4 and the right one is stage 1 ~ 3 and 4S). (D) The MYCN subgroup analysis of RPI in the training cohort (The left one is amplified and the right one is not amplified). [file Image_1.TIF]

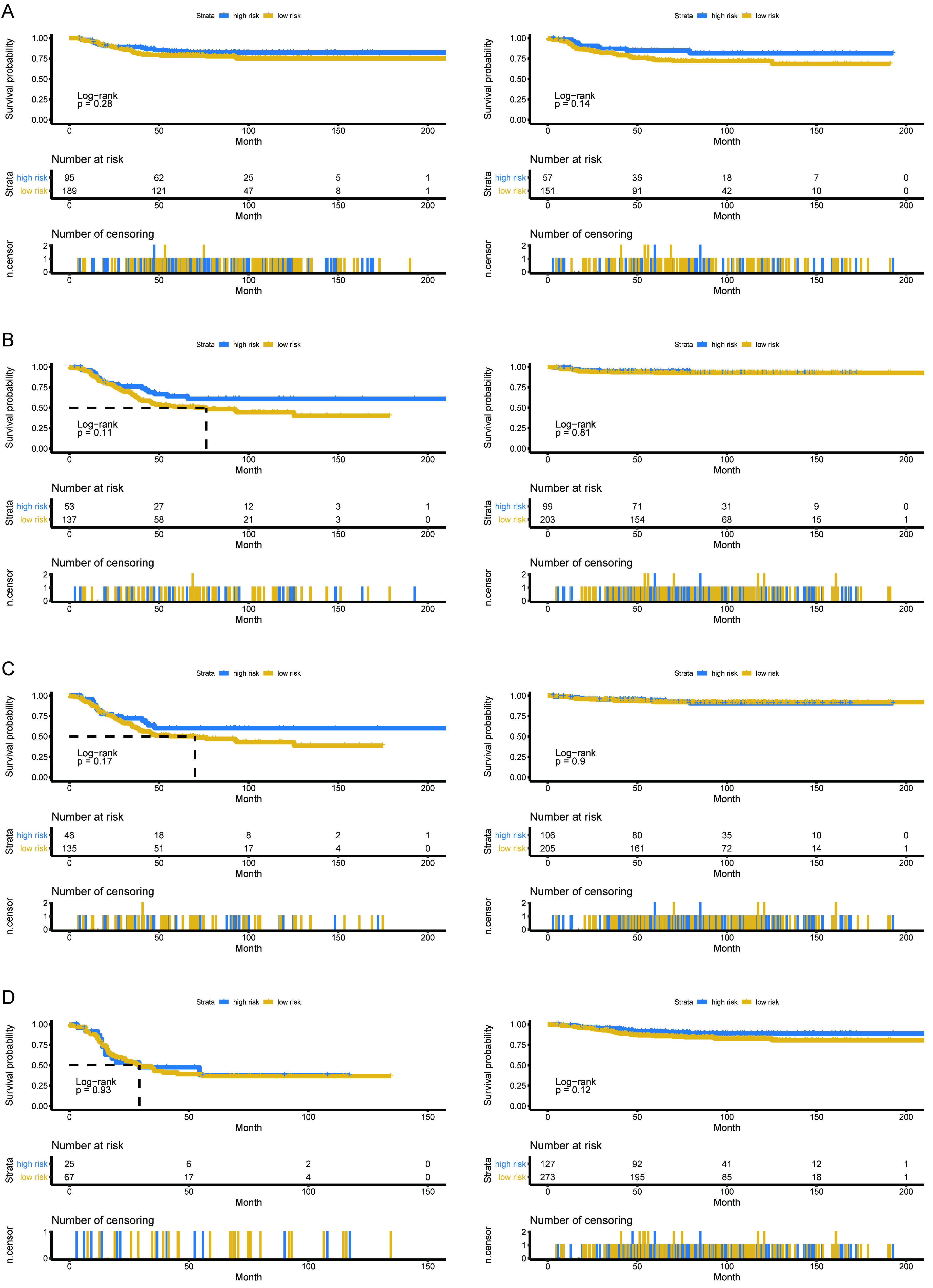

Supplement: Figure S2 — The subgroup analysis of RPI in the validation cohort. (A) The gender subgroup analysis of RPI in the training cohort (The left one is male and the right one is female). (B) The age subgroup analysis of RPI in the training cohort (The left one is ≥ 18 months and the right one is < 18 months). (C) The stage subgroup analysis of RPI in the training cohort (The left one is stage 4 and the right one is stage 1 ~ 3 and 4S). (D) The MYCN subgroup analysis of RPI in the training cohort (The left one is amplified and the right one is not amplified). [file Image_2.TIF]
